# Clinical Characteristics and Prognosis of Renal Cell Carcinoma With Spinal Bone Metastases

**DOI:** 10.3389/fonc.2021.659779

**Published:** 2021-06-16

**Authors:** Jianpo Zhai, Ning Liu, Hai Wang, Guanglin Huang, Libo Man

**Affiliations:** Department of Urology, Beijing Jishuitan Hospital, Beijing, China

**Keywords:** renal cell carcinoma, spinal bone metastasis, prognosis, en bloc resection, characteristic

## Abstract

**Background:**

The prognosis of renal cell carcinoma (RCC) with spinal bone metastasis (sBM) varies greatly. In this study, we aimed to define the clinical characteristics and prognostic factors of RCC with spinal bone metastasis (sBM) in our center.

**Methods:**

The clinical and medical records of RCC patients with sBMs were collected. The gender, age, time of BM, the extent of BM, the number of BMs, the presence or absence of visceral metastasis, and the pathological type of BM were investigated. All patients were followed up regularly. Overall survival (OS) was calculated from the date of BMs diagnosis to death or last follow-up using Kaplan-Meier method and modelled with Cox regression analysis.

**Results:**

Forty-three RCC patients with sBM were collected. sBM was found synchronously in 30 patients (70%) and metachronously in 13 patients (30%). The median survival time was 30 months in 13 patients (30%) with solitary sBM and 19 months in 30 patients (70%) with multiple sBMs (*P* = 0.002). Visceral metastasis occurred in 12 patients (28%) with the median survival time of 17 months, while the other 31 patients (72%) had no visceral metastasis with the median survival time of 29 months (*P*<0.001). En-block resection was done in 10 patients with median survival time of 40.1 months. Non-en-block resection were done in 33 patients with median survival time of 19.7 months (*P*<0.001). Multivariate COX regression analysis showed that MSKCC score, number of BM, visceral metastasis, and en-block resection are the independent prognosis factors of RCC patients with sBM.

**Conclusions:**

MSKCC risk stratification, number of sBM, visceral metastasis and en-block resection are significant prognostic factors for OS in RCC patients with spinal BM. Therefore, for selected patients who has solitary spinal BM with no visceral metastasis, en-block resection of spinal BM can potentially prolong survival and is the treatment of choice.

## Introduction

Renal cell carcinoma (RCC) is a common urinary system malignant tumor that accounts for 2–3% adult malignant tumors. The male-female ratio was approximately 1.5:1 and the age group with the highest prevalence was 50–70 years (1, 2). The incidence of RCC has increased by an average of 2% per year (3, 4). One third of patients with RCC have metastatic lesions at the time of diagnosis, and 20–40% develop metastatic lesions following radical or partial nephrectomy (5–7). The bone is the second most common site of RCC metastasis, and approximately 40% of these occur in the spine. Spinal bone metastasis (sBM)is considered a negative prognostic factor for RCC (8). Encouragingly, with present immunotherapy/immunotherapy and immunotherapy/TKI, the proportion of patients achieving an objective response has been between 30 and 60%, with complete responses in 7–10% of patients (9, 10). However removal of BM provides the only potentially curative treatment and prolongs the survival time of RCC with BM. So it is important to identify the prognostic factors that can be useful in guiding clinical decisions to improve survival in this patient population.

## Material and Methods

### Patient Selection

A total of 43 RCC patients with spinal bone metastasis were collected. They were admitted to the Department of Urology, Bone Oncology and Spine Surgery Beijing Jishuitan Hospital from 2009 to 2019. The clinical and medical records were investigated. The patients were followed up regularly. Inclusion criterion: patients with (1) newly diagnosed sBM from RCC (2), sBM diagnosed by bone scan or PET-CT (3), definite pathology diagnosis of the sBM. Exclusion criterion: patients with (1) concomitant other malignant neoplasms (2), no surgical treatment of bone metastasis (3), target therapy or radiotherapy.

### Investigations and Follow-Up

The gender, age, time of BM, the extent of BM, the number of BMs, the presence or absence of visceral metastasis, and the pathological type of BM were investigated. All patients were followed up regularly after surgery with 3 months interval in the first year and 6 months interval thereafter. Postoperative surveillance included routine clinical and laboratory examinations every third month, computed tomography scans of the chest and abdomen every third month. Patients were followed by their physician until the patient’s death or date of the last documented contact.

According to the time of BM, patients were divided into two groups: RCC with sBM synchronously group and RCC with sBM metachronously group. According to the extent of BMs, patients were divided into five groups: only sBM, sBM with limb BMs, sBM with ribs or clavicle BMs, sBM with pelvis or iliac BMs, multiple sites BMs. Using the MSKCC criteria (Karnofsky performance status <80%, interval from diagnosis to systemic treatment <1 year, hemoglobin < lower limit of normal, corrected calcium >10 mg/dl/> 2.5 mmol/L, LDH >1.5× upper limit of normal), favorable risk is defined as zero poor prognostic factor, intermediate risk is 1–2 poor prognostic factors, and poor risk is more than two poor prognostic factors.

### Statistical Analysis

Statistical software SPSS 20.0 was used to process data. The measurement data was expressed in M(range) and the count data was expressed in quantity and percentage. OS was calculated from the date of BMs to death or last follow-up. Kaplan-Meier method and Mantel-Haenszel log-rank test was used to compare survival among groups. A Cox regression model was applied to the data with a univariate and multivariate approach. Variables not fitting at univariate regression analysis were excluded for the multivariate model. The difference between groups was determined by Lon-rank test. The difference was statistically significant at P < 0.05.

## Results

Forty-three newly diagnosed RCC patients with sBM were collected (**Table 1**). Among them 35 were male (accounting for 81%) and 8 were female (accounting for 19%). The ratio of male to female patients was 4.38:1. The youngest was 45 years old, the oldest was 82 years old, and the median age was 58 ± 6.8 years. Prognostic group using MSKCC criteria was good in 7 (16%), intermediate in 28 (65%), and poor in 8 (19%). The most common histological subtype was clear-cell RCC (39 patients, 91%). None of the patients received systemic therapy.

**Table 1:** Clinical characteristics of newly diagnosed RCC with spinal BM.

Variable		Patients, n (%), (N = 43)
Median age, y (range)		58 (45–82)
Gender		
Male		35 (81)
Female		8 (19)
MSKCC criteria		
Good		7 (16)
Intermediate		28 (65)
Poor		8 (19)
Fuhrman grade		
2		15 (35)
3		21 (49)
4		7 (16)
Time of sBM		
Synchronously		30 (70)
Metachronously		13 (30)
Number of sBM		
Solitary		13 (30)
Multiple		30 (70)
Extent of sBM		
Only sBM		13 (30)
sBM with limb BMs		10 (23)
sBM with ribs or clavicle BMs		4 (9)
sBM with pelvis or iliac BMs		12 (29)
Multiple sites BMs		4 (9)
Visceral Metastasis		
Yes		12 (28)
No		31 (72)
Resection of BMs		
En-block		10 (23)
Non-en-block		33 (77)
Tumor histology		
Clear Cell Carcinoma		39 (91)
Non-clear Cell Carcinoma		4 (9)

**Figure 1:**
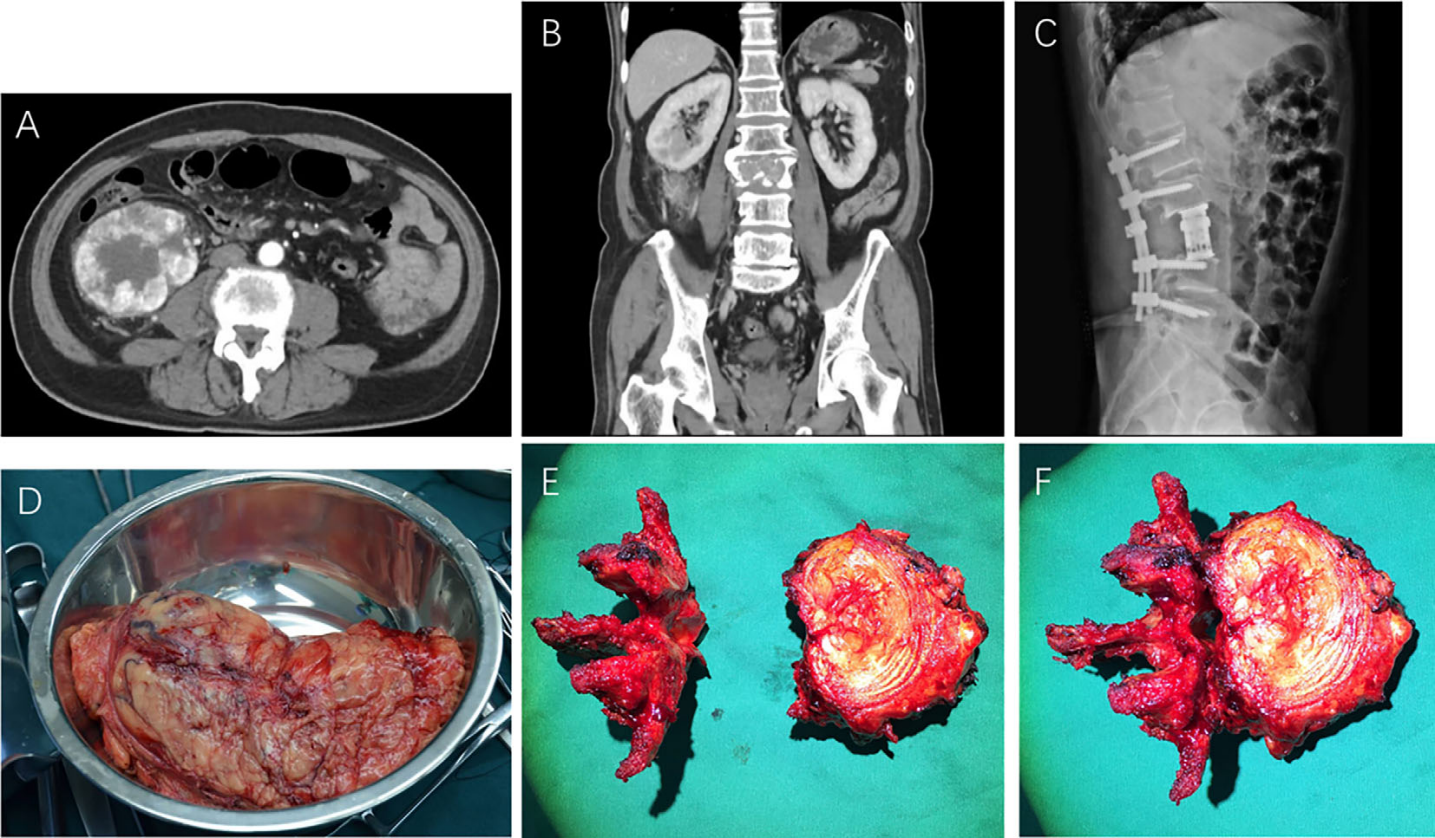
Preoperative CT scans **(A, B)**, postoperative X-ray **(C)**, resected primary renal cell carcinoma specimen **(D)**, and en-block resected metastatic vertebra **(E, F)** of RCC with spinal bone metastasis.

sBM was found synchronously in 30 patients (70%), as shown in **Figures 1A, B**, and metachronously in 13 patients (30%). In RCC patients with sBM metachronously, the shortest time for the occurrence of sBM was 3 months, the longest time was 108 months, and the median time was 14.2 months. With regards to the number of sBMs, 13 patients (30%) had solitary sBM and 30 patients (70%) had multiple sBMs. In terms of the extent of sBM,13 patients (30%) had the only sBM, 10 patients (23%) had sBM with limb BMs. 4 patients (9%)had sBM with ribs or clavicle BMs. 12 patients (29%) had sBM with pelvis or iliac BMs. Four patients (9%) had multiple sites BMs. Visceral metastasis occurred in 12 patients (28%), while the other 31 patients (72%) had no visceral metastasis. The most common visceral metastasis was lung metastasis in 7 patients (58.3%) followed by the adrenal metastasis in 3 patients, and liver metastasis in 2 patients. In addition, 10 patients (23%) received the radical nephrectomy and en-block resection of spinal BM, as shown in **Figures 1C–F**.

**Figure 2:**
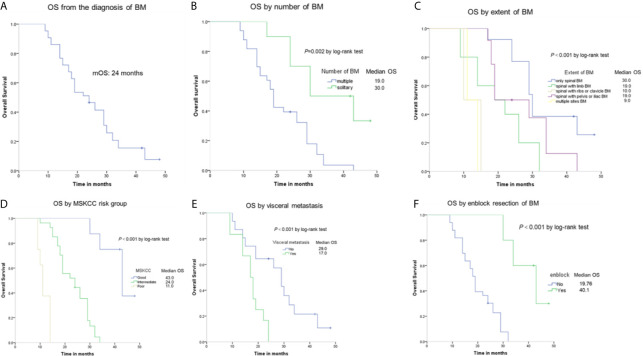
Kaplan-Meier estimates of overall survival (OS) among RCC patients with spinal BM **(A)** and the OS according to number of sBM **(B)**, extent of sBM **(C)**, MSKCC risk group **(D)**, visceral metastasis **(E)**, and en-block resection **(F)**.

Forty-three patients were followed up for 9–48 months, with a median follow-up of 23 months. A total of 37 patients (86.4%) died at the last follow-up. Of the 31 patients without visceral metastasis when diagnosed, 17 patients had the lung metastasis and 5 patients had the liver metastasis during the follow-up. The median overall survival time of the patients was 24 months, as shown in **Figure 2A**. Patients with a solitary BM had a longer survival than patients with multiple BMs (30 *vs* 19 months, respectively, *P* = 0.002), as shown in **Figure 2B**. Moreover, the extent of BM had impact on OS (**Figure 2C**). The median survival time was 30 months in the only sBM group, 19 months in the sBM with limb BMs group, 10 months in the sBM with ribs or clavicle BMs group, 19 months in the sBM with pelvis or iliac BMs group, and 10 months in the multiple sites BMs group respectively. The difference between these groups was statistically significant (*P*<0.001). Patients with good MSKCC risk score had the longest OS than those with intermediate and poor MSKCC risk score (43 *vs* 24 *vs* 11 months, respectively, *P*<0.001), as shown in **Figure 2D**. The visceral metastasis was significantly associated with OS (17 *vs* 29 months, respectively, *P*<0.001), as shown in **Figure 2E**. Significant differences in terms of OS were found when comparing patients undertaking en-block resection of BM with those of non-en-block resection (40.1 *vs* 19.76 months, respectively, *P*<0.001), as shown in **Figure 2F**.

Multivariate COX regression analysis showed that MSKCC score, number of BM, visceral metastasis, and en-block resection are the independent prognosis factors of RCC patients with sBM, as shown in **Table 2**. MSKCC score was associated with OS (*P* = 0.006). Patients with multiple BMs had shorter survival (*P* = 0.035). In addition, visceral metastasis remained a negative prognostic factor for OS (*P* = 0.026). With regards to the therapy of BM, en-block resection of BM was associated with a longer survival (*P* = 0.015).

**Table 2:** COX multivariate analysis for overall survival of RCC with spinal BM.

Covariate	*P* value	HR (95% CI)
MSKCC group	0.006	0.512 (0.35–0.68)
Time of sBM	0.554	1.331 (0.229–2.205)
Fuhrman Grade	0.293	1.011 (0.932–1.096)
Number of sBMs	0.035	1.302 (0.345–4.919)
Extent of BMs	0.327	1.183 (0.845–1.656)
Visceral Metastasis	0.026	4.322 (1.443–12.947)
En-block Resection	0.015	0.192 (0.032–1.139)

## Discussion

The prognosis of RCC with sBM varies greatly. Several prognostic variables, including MSKCC, have been identified to predict survival in metastatic RCC (11–13). The present study focused mainly on the prognostic factors associating with the BM, such as the number of sBM, the extent of sBM, and the surgical approach of sBM.

The number and extent of BMs are closely related to the prognosis of mRCC patients (14, 15). In the present study, patients with a solitary BM had a longer survival than patients with multiple BMs (30 *vs* 19 months, respectively, *P* = 0.002). This finding is consistent with a previous study from Ruatta’s (16). In addition, our results indicate that the more extensive of BM, the worse of the survival of the patient. The median survival time was 30 months in the only spinal BM group, and 10 months in the multiple sites BMs group (*P*<0.001), that is consistent with previously reported data. A retrospectively study by Tatsui showed that patients whose spine was the only site of metastasis had a median OS of 19 months after surgery, significantly longer than the 9.7 months observed in patients with additional extraspinal metastasis sites (p < 0.001) (17). Haruki and Fukushima’s study also found that the extent of BM affects patients’ prognosis (18, 19). The poor prognosis of the RCC with extensive BM is possibly due to the poor performance status of patients and the incomplete resections of all the BMs.

Visceral metastasis such as liver metastasis, lung metastasis are the poor prognostic factor for the RCC with sBM (14, 20). In the study, the median survival time was 17 months and 29 months respectively in patients with visceral metastases and those without visceral metastases (P<0.001). This result is consistent with other reports. Yutaka investigated 50 RCC patients with BM. The univariate and multivariate Cox regression analysis indicated that visceral metastasis was an independent unfavorable prognostic factor (21). Ruatta took a large scale single center prognosis study on 300 patients with BM from RCC and found that the OS of patients with visceral metastasis was significant shorter than patients without visceral metastasis (17.6 months *vs* 46.4 months, P < 0.0001) (16). Therefore, the prognosis of RCC patient with visceral metastasis is very poor. For these patients, palliative surgery and symptomatic treatment, which can improve the quality of life, should mainly be recommended.

The surgical approach of BM affects the prognosis of RCC patients with BM (22, 23). Langerhuizen assessed the local cancer recurrence rate between different surgical treatments for BM in 183 patients. The results showed that the recurrence rate was 39% after stabilization only, 22% after intralesional curettage, and 12% after metastasectomy (P = 0.003). Patients who received metastasectomy had better survival (P = 0.020) (24). These results were also confirmed by SATOSHI KATO’s study. The estimated median CSS time was 130 months in the 36 RCC patients who received nephrectomy and complete resection of solitary spinal lesion, with the 3, 5, and 10 years CSS rates 77.8, 69.1, and 58% respectively (25). In our study, 10 patients underwent en-block resection of the BM, with a median survival time of 40.1 months. Thirty-three patients underwent non-en-block resection of the BM, with a median survival time of 19.76 months (*P*<0.001). En-block resection of the sBM can prolong the survival time of RCC patient. However, only a few centers in our country can perform such complicated surgery due to the higher operative difficulty and incidence of vascular injury. Along with the improvements in surgical techniques and preoperative embolization, en-block spondylectomy has achieved excellent clinical results with low morbidity (26–28).

The present study initially analyzed the prognostic factors of RCC with sBM. However, some limitations should be acknowledged. These include the small and heterogeneous series of patients in the single center, and the retrospective data collection, which can introduce bias and errors. Furthermore, the length of follow-up did not allow to fully justify the conclusion on overall survival (OS). Moreover, the prospects of comorbidity were not embraced in this study. Despite these limitations, the results of the study are informative. The results of this study show that MSKCC risk stratification, number of sBM, visceral metastasis, and en-block resection are independent significant prognostic factors for OS in RCC patients with spinal BM. Therefore en-block resection of spinal BM is one of treatment of choices for the metastatic RCC patients who have solitary spinal BM, with no visceral metastasis. Certainly, further improvement in treatment modalities to cure BM thereby decreasing morbidity and mortality was needed.

## Conclusions

In conclusion, MSKCC risk stratification, number of sBM, visceral metastasis, and en-block resection are significant prognostic factors for OS in RCC patients with spinal BM. Therefore, for selected patients who have solitary spinal BM with no visceral metastasis, en-block resection of spinal BM can potentially prolong survival and is the treatment of choice.

## Data Availability Statement

The original contributions presented in the study are included in the article/supplementary material. Further inquiries can be directed to the corresponding author.

## Ethics Statement

The study was approved by the ethics committees of the Beijing Jishuitan Hospital. The patients/participants provided written informed consent to participate in this study.

## Author Contributions

JZ and NL contributed to the data collection. JZ and HW contributed to the analysis writing. JZ and NL contributed to the editing and submission of the article. GH and LM contributed to the conception of the project and editing of the article. All authors contributed to the article and approved the submitted version.

## Funding

This work was supported by the Beijing Jishuitan Hospital Nova Program (Code: XKXX201616) and Beijing Hospitals Authority Youth Programme (Code: QML20170405).

## Conflict of Interest

The authors declare that the research was conducted in the absence of any commercial or financial relationships that could be construed as a potential conflict of interest.
